# The Efficacy of Wrestling-Style Compression Suits to Improve Maximum Isometric Force and Movement Velocity in Well-Trained Male Rugby Athletes

**DOI:** 10.3389/fphys.2017.00874

**Published:** 2017-11-28

**Authors:** Daniel T. McMaster, Christopher M. Beaven, Brad Mayo, Nicholas Gill, Kim Hébert-Losier

**Affiliations:** ^1^Health, Sport and Human Performance, University of Waikato, Hamilton, New Zealand; ^2^Bay of Plenty Rugby, Bay of Plenty, New Zealand; ^3^Sports Performance Research Institute New Zealand, Auckland University of Technology, Auckland, New Zealand

**Keywords:** compression, isometric strength, horizontal force, countermovement jump, sled push, rugby athletes

## Abstract

**Purpose:** The prevalence of compression garment (CG) use is increasing with athletes striving to take advantage of the purported benefits to recovery and performance. Here, we investigated the effect of CG on muscle force and movement velocity performance in athletes.

**Methods:** Ten well-trained male rugby athletes wore a wrestling-style CG suit applying 13–31 mmHg of compressive pressure during a training circuit in a repeated-measures crossover design. Force and velocity data were collected during a 5-s isometric mid-thigh pull (IMTP) and repeated countermovement jump (CMJ), respectively; and time to complete a 5-m horizontal loaded sled push was also measured.

**Results:** IMTP peak force was enhanced in the CG condition by 139 ± 142 N (effect size [ES] = 0.36). Differences in CMJ peak velocity (ES = 0.08) and loaded sled-push sprint time between the conditions were trivial (ES = −0.01). A qualitative assessment of the effects of CG wear suggested that the likelihood of harm was unlikely in the CMJ and sled push, while a beneficial effect in the CMJ was possible, but not likely. Half of the athletes perceived a functional benefit in the IMTP and CMJ exercises.

**Conclusion:** Consistent with other literature, there was no substantial effect of wearing a CG suit on CMJ and sprint performance. The improvement in peak force generation capability in an IMTP may be of benefit to rugby athletes involved in scrummaging or lineout lifting. The mechanism behind the improved force transmission is unclear, but may involve alterations in neuromuscular recruitment and proprioceptive feedback.

## Introduction

The use of compression garments (CG) has become increasingly popular among athletes, looking to enhance performance, reduce fatigue, and decrease injury risk during training and competition (Gill et al., 2006; Born et al., 2013). There is some scientific evidence to suggest that CG may improve athletic performance by affecting blood flow (Bochmann et al., 2005) and oxygen kinetics (Bringard et al., 2006; Coza et al., 2012); applying localized pressure to help stabilize and support underlying tissues, in turn increasing force production (Kraemer et al., 1998); and reducing fatigue (MacRae et al., 2011; Hamlin et al., 2012; Born et al., 2014). Specifically, research indicates that lower body CGs may have *trivial* to *small* positive effects on maximal effort jump and sprint performance in trained athletes (Kraemer et al., 1996; Doan et al., 2003; Duffield and Portus, 2007; Duffield et al., 2010; Born et al., 2013; Wannop et al., 2016). Positive effects observed during dynamic movements wearing lower body CG have been attributed to enhanced power maintenance (Kraemer et al., 1996) biomechanical changes (Doan et al., 2003; Born et al., 2014), and a reduced perception of effort (Faulkner et al., 2013).

The amount of pressure applied to the body or body part is determined by the physical and mechanical properties of the CG material and the fit of the material relative to the athlete's anthropometry and morphology. Commercially available CGs may be composed of spandex, nylon, neoprene, polyurethane, elastane, and/or rubber, which provide varying amounts of pressure. Whole body as opposed to lower body only CG that apply pressure over a larger surface area of the body may have a larger positive effect on jump and possibly sprint performance (Duffield and Portus, 2007). In fact, combined upper and lower body CG have been reported to increase kinetic and kinematic variables during powerlifting (Blatnik et al., 2012; Godawa et al., 2012).

Minimal research has investigated the effects of wrestling-style CG on horizontal and vertical force production capabilities in athletes in which the upper legs and portions of the trunk are compressed. Therefore, the aims of this investigation were to determine the effects of wearing a wrestling-style compression suit on repeated vertical countermovement jumps, a horizontal loaded sled push and isometric mid-thigh pull performance in well-trained rugby athletes.

## Methods

### Experimental approach and design

Using a repeated-measures crossover design, all athletes performed a high force-high velocity circuit on two separate occasions (7 days apart), once with and once without wearing the wrestling-style CG. During the non-compressed control session (CON), athletes wore loose fitting training attire (e.g., rugby shorts and a singlet or T-Shirt). Footwear was matched across conditions with both sessions occurring at the same time of day.

### Subjects

The participants comprised of 10 well-trained male rugby players (age = 21.0 ± 2.6 y, height = 1.83 ± 0.07 m, body mass = 95.7 ± 10.8 kg; one-repetition maximum parallel back squat = 167 ± 16 kg). The University of Waikato Human Research Ethics Committee granted study approval and each athlete provided written informed prior to partaking in the study.

### Compression garment

**Figure 1:**
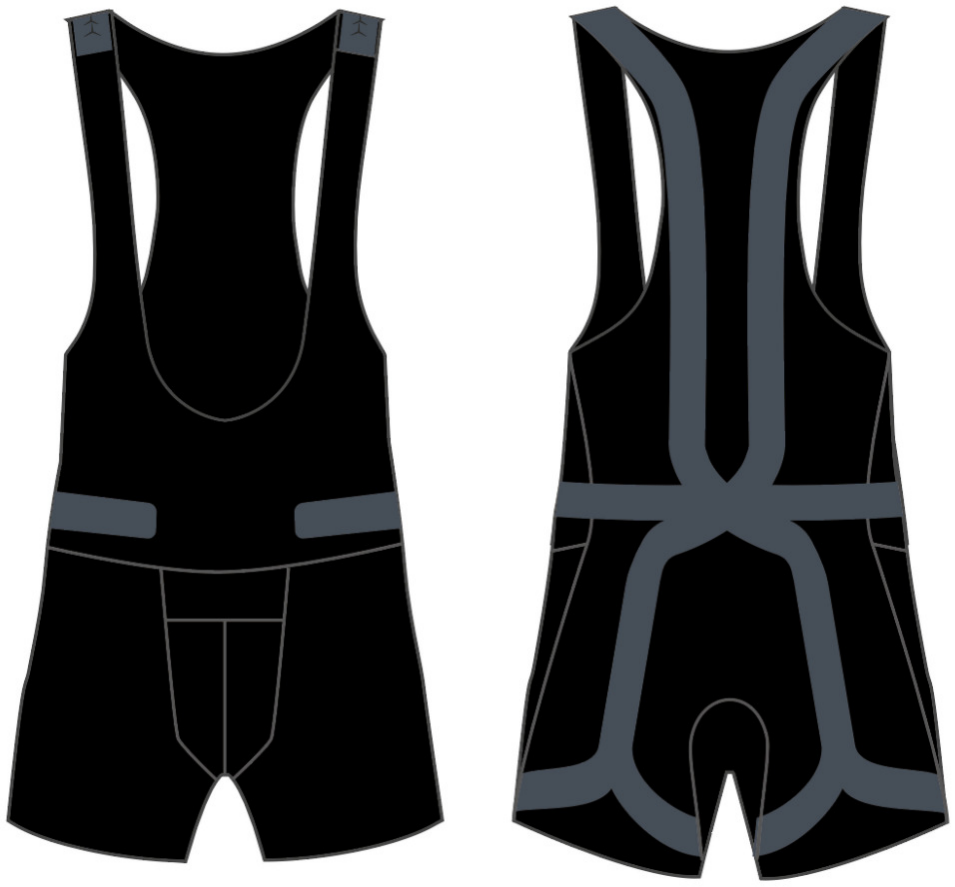
Schematic diagram of the wrestling style compression garment.

The wrestling-style CG suit (Chassis, Adidas, Herzogenaurach, Germany) used in this study was similar to the CG suits that wrestlers and Olympic weightlifters wear (Figure 1). The CG ran from just above the knee, up the back, over the scapula, and down the right and left pectoralis major muscles with the neck, chest, and upper abdomen uncovered. The garment material is made to be light, strong, and compressive, and consists of 65% polyamide and 35% elastane. Additionally, the garment has strips of criss-crossing rubber on the inner surface, creating a double layer of rubber designed to maximize compression and increase tension. There is also a sticky inner surface running along the shoulder straps designed to prevent the garment from sliding on the skin surface. The amount of pressure applied by the CG at the point of maximal mid-thigh girth standing stationary in anatomical position ranged from 13 to 31 mmHg (i.e., smallest to largest athlete) as measured with the Kikuhime pressure-monitoring device to the nearest 1 mmHG (MediGroup, Melbourne, Australia). The same trained physiotherapist performed three pressure measurements to ensure consistency in readings and care was taken to avoid the garment seams.

### Procedures

Following a brief dynamic warm-up consisting of dynamic stretches and one round of the circuit at sub-maximal effort (~50%), participants performed three maximal effort (i.e., with intent to produce maximal force and/or maximum velocity) rounds of the experimental circuit in sequential order (Table 1). The circuit consisted of five repeated vertical countermovement jumps (CMJ) using a lightweight dowel placed across the athlete's upper trapezius, a 5-m horizontal loaded-sled push (i.e., prowler sprint) using a load of 75 kg, a 5-s maximum isometric mid-thigh pull (IMTP), five barbell bench press repetitions using a load of 50 kg and five prone bench pulls using a load of 50 kg. Each athlete was given 110–115 s rest between each exercise (i.e., the circuit was structured in that each athlete performed each successive exercise of the circuit 2 min following the start of the preceding exercise).

**Table 1:** Experimental circuit timing.

**No**.	**Exercise**	**Reps/ distance/time**	**Exercise duration**	**Inter-exercise rest**	**Total duration**
1	Countermovement jumps	5 reps	~10 s	~110 s	2 min
2	Horizontal sled push (75 kg)	5 m	~5 s	~115 s	2 min
3	Isometric mid-thigh pull	5 s	5 s	~115 s	2 min
4	Barbell bench press (50 kg)	5 reps	~10 s	~110 s	2 min
5	Barbell prone bench pulls (50 kg)	5 reps	~10 s	~110 s	2 min
Repeat the circuit 3 times					30 min

CMJ peak velocity was measured at a sampling frequency of 50 Hz using a linear position transducer (Gymaware, Kinetic Performance Technology, Canberra, Australia) placed on the floor and attached to the dowel laterally on the left-hand side of athletes. Horizontal loaded sled push sprint times were measured using a motion start device and single beam infrared timing light set to a height of 0.73 m (TC-Timing System, Brower, Draper, Utah, USA). The front of the sled was placed on the 5-m sprint start line with the athlete standing stationary in a split stance position. The start time was triggered by the motion-sensing start device placed adjacent to the athlete's rearfoot. IMTP peak force was measured using two dual axis portable force plates (Dual-Axis Force Platform, PASCO, Roseville, California, USA) sampled at a frequency of 500 Hz. No measurements were collected for the bench press or bench pull.

Following the second session, all athletes completed a 3-point perceptual rating of the effect of CG on vertical jump, horizontal sled push and IMTP performance. The rating scale was as follows: 1—CG had a positive effect on my performance, 2—CG had no effect on my performance, and 3—CG had a detrimental effect on my performance.

### Statistical analysis

Means and standard deviations (mean ± SD) were used to represent centrality and spread of the data. Data were analyzed for practical significance using p-values (P ≤ 0.10) and magnitude-based inferences (90% confidence intervals). Effect size calculations (ES = Mean_CG_–Mean_CON_/SD_pooled_) were used to represent the standard differences in performance and were interpreted as trivial (*ES* < 0.20), small (*ES* = 0.20–0.60), moderate (*ES* = 0.60–1.20), and large (*ES* ≥ 1.20; Hopkins, 2010). The likelihood of the CG having a beneficial, negligible, or harmful effect on performance was qualitatively assessed based on magnitude-based inferences (<1%, almost certainly not; 1–5%, very unlikely; 5–25%, unlikely; 25–75%, possibly; 75–95%, likely; 95–99%, very likely; >99%, almost certainly). Spaghetti plots were constructed to illustrate the individual responses to CG.

## Results

**Figure 2:**
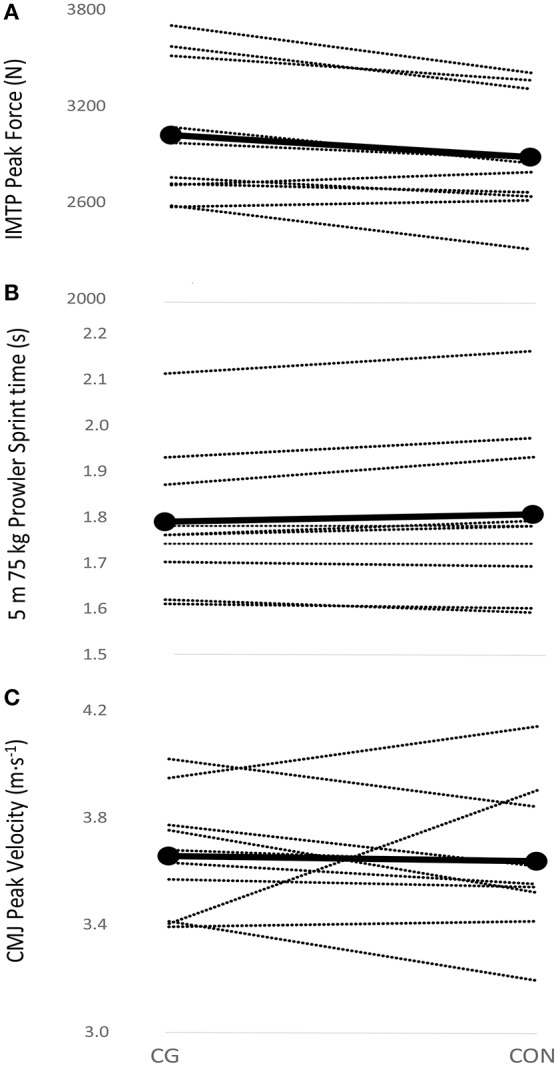
Spaghetti plot of performance reponses to compression garment use in: **(A)** isometric mid-thigh pull (IMTP); **(B)** horizontal loaded sled push; and **(C)**, countermovement jump (CMJ).

A *small* and *likely beneficial* effect (ES = 0.36) of CG on IMTP peak force production was observed; whereas *trivial* and *unlikely harmful* effects of CG wear on horizontal sled push and CMJ performance were observed (Tables 2, 3). The individual data for each athlete with and without CG are presented in Figures 2A–C. Half of the athletes felt that the CG had a positive effect on IMTP and CMJ, while no athlete felt that the CG positively affected prowler sprint performance. No athlete perceived the CG as having a detrimental effect on performance.

**Table 2:** Comparison between wrestling-style compression suit and non-compressive clothing on isometric mid-thigh pull, vertical jump and horizontal sled push performance.

	**Compression**	**No Compression**	***%Diff***	***P***	***ES***
	**Mean ± SD**	**Mean ± SD**			
IMTP peak force (N)	2, 938 ± 426	2, 799 ± 352	4.8	0.013	0.36
CMJ peak velocity (m·s^−1^)	3.34 ± 0.21	3.33 ± 0.20	0.5	0.719	0.08
5 m 75 kg sled push (s)	1.85 ± 0.21	1.85 ± 0.16	0.1	0.962	−0.01

*%Diff, Percentage difference; CMJ, Countermovement jump; ES, Effect size; IMTP, Isometric mid-thigh pull; P, Statistical significance set at P ≤ 0.10*.

**Table 3:** Likelihood of the wrestling-style compression suit having a beneficial, negligible or harmful effect on performance^*^.

	**Beneficial**	**Negligible**	**Harmful**
IMTP peak force (N)	90%	10%	0%
	Likely	Unlikely	Most unlikely
CMJ peak velocity (m·s^−1^)	30%	59%	11%
	Possibly	Possibly	Unlikely
5 m 75 kg sled push (s)	17%	65%	19%
	Unlikely	Possibly	Unlikely

* *Likelihood effects were based on a sample size of n = 10, 90% confidence limits and a threshold effect size of 0.20. CMJ, countermovement jump; IMTP, isometric midthigh pull*.

## Discussion

The wrestling-style CG suit had a likely beneficial effect on maximum isometric strength (peak force), but no meaningful effect on vertical countermovement jump (peak velocity) or resisted horizontal sprint (5-m time) performance in well-trained male rugby athletes. These findings are suggestive of an improved ability to transmit force maximally. Despite half of the athletes perceiving that the CG positively affected their vertical CMJ jump performance; the objective CMJ measures indicate that no such effect was present.

The improved ability to produce peak force in the isometric mid-thigh pull may have important implications for rugby athletes engaged in on-field scrummaging although similar peak power production has been reported between compressed and non-compressed suits in a scrum machine effort (Duffield et al., 2008). The improved ability to produce force isometrically might also benefit lineout lifters at lift initiation and at the zenith of the lineout where isometric lower-body strength are required. The underlying mechanisms of producing muscle force include central and peripheral excitability of the motor pathway (i.e., a series of electrochemical processes), where optimal output from the primary motor cortex results in increased motoneuron firing rates and increased motor unit recruitment patterns (Tax and Denier van der Gon, 1991; Hodson-Tole and Wakeling, 2009; Moscatelli et al., 2016). Compression has been reported to impact muscle architecture (Wakeling et al., 2013), muscle activity (Nigg and Wakeling, 2001; Hsu et al., in press), and may have the ability to stimulate cutaneous mechanoreceptors to facilitate motoneuron control (Iles, 1996). Indeed, changes in neuromuscular function have previously been attributed to enhanced tactile input that alter the excitability of the central nervous system and modulate proprioceptive afferent feedback loops (Karlsson and Andreasson, 1992). However, the precise mechanisms for the observed isometric force improvement requires further investigation.

A systematic review of research has indicated that CGs may have beneficial effects on high impulse movements utilizing the stretch-shortening cycle, such as jumping and sprinting (Born et al., 2013); however, many researchers have been unable to demonstrate an effect of CG on maximal sprint performance in athletes (Doan et al., 2003; Duffield et al., 2008, 2010; Higgins et al., 2009; Houghton et al., 2009; Born et al., 2014). Current findings indicate that CGs were unlikely to have beneficial (or harmful) effects on horizontal sled push performance in well-trained male rugby athletes. It has been suggested that the elasticity supplied by CG may increase leg acceleration in the terminal swing phase of sprinting; however, the compression supplied by the CG may also inhibit range of motion during hip flexion (Doan et al., 2003; Bernhardt and Anderson, 2005; Born et al., 2014). This resistance may contribute to the negligible effects of CG wear on both resisted and non-resisted maximal sprints and accelerations, including the 5-m horizontal sled sprint protocol.

Similarly, the current findings along with previous studies have been unable to demonstrate a positive effect of CGs on maximal jump performance despite evidence to suggest that lower body power is enhanced (Kraemer et al., 1998; Ali et al., 2011; Rugg and Sternlicht, 2013). Kraemer et al. (1998) attributed improved resilience to fatigue profiles seen through enhanced mean power output from repetitive CMJ to decreased muscle oscillation and enhanced joint-position sense. An earlier study by Kraemer et al. (1996) reported similar findings in volleyball athletes with an improvement in mean power during 10 repeated countermovement jumps when wearing compression shorts. Kraemer et al. (1996) also noted a sex interaction effect whereby maximal force production was enhanced in males but not in females with CG shorts. Doan et al. (2003) were able to detect a 0.024 m increase in a single-repetition maximal CMJ performed by male and female collegiate track athletes wearing a custom-fit compressive garment, although this difference was not apparent when the sexes were analyzed separately. The authors attributed the improved jump performance to a significantly deeper squat depth and enhanced propulsive impulse.

The individual responses to CG noted herein for CMJ performance (see Figure 1C) have been observed elsewhere. Wannop et al. (2016) demonstrated using a range of different compression and stiffness garments, that 90% of athletes performed their best jump wearing a compression garment, with 60% also performing their worst jump. Thus, the athletic response to the magnitude of compression is highly individualized and the factors relating to the beneficial or detrimental impact of CG on dynamic movements are not well understood. While a minimum level of compression of 15.1 mmHg at the thigh has been reported to be required to improve venous blood flow (Watanuki and Murata, 1994), a lack of relationship between anthropometric characteristics and the magnitude of compressive force has also been reported (Hill et al., 2015). This poor relationship suggests that there is a complex interaction between the compression garment and individual body shapes, reinforcing the requirement for individually tailored garments. We acknowledge that the garments used herein were not custom fitted to each individual athlete tested, and that there was the potential for a placebo effect as there was no blinding of the participants to the intervention. While MacRae et al. (2011), have noted that any suggestion of an optimal compression pressure is currently “unjustified,” it was clear that our athletes experienced a range of magnitudes of compression (13–31 mmHg). We also acknowledge that there is the potential for aspects other than strictly compression to influence the performance measures, including skin temperature (Duffield and Portus, 2007), biomechanical changes (Born et al., 2014), and proprioception (Bernhardt and Anderson, 2005).

## Conclusion

From a practical perspective, the findings from the present study provide strength and conditioning practitioners with evidence that the use of a wrestling-style compression suit can likely provide athletes with some degree of structural and/or neuromuscular improvement in force capabilities in well-trained rugby athletes. The relationship of this improvement to multi-joint maximum strength testing and training (e.g., squat, deadlift, clean, and snatch), as well as on-field performance, is currently unclear. In contrast, the compression suits provided no consistent benefit to resisted horizontal sprint acceleration performance or CMJ performance. Appropriately constructed and fitted compression suits might offer additional benefits for other force dominant tasks in sport, such as scrummaging, lifting, rucking, tackling, and changing direction. While this view is plausible, it requires further scientific confirmation.

With the application of localized compression, an athlete may feel more stabilized and supported while performing sprints, jumps, pushing, and lifting movements. Athlete morphology and anthropometric characteristics have the potential to influence the amount of localized pressure provided by the CG and in turn affect performance. Therefore, further investigating what constitutes “correct” individualized CG sizing is recommended to ensure that CG provide an appropriate amount of localized pressure to maximize athlete performance.

## Ethics statement

The study was approved by the University of Waikato Human Research Ethics Committee according to the Helsinki Declaration guidelines. Participants were fully informed and signed a consent form that indicated they could withdraw from the study at any time.

## Author contributions

DM, CB, BM, NG: Substantial contributions to the conception or design of the work; DM, CB, BM, KH-L: The acquisition, analysis, or interpretation of data for the work; DM, CB, BM: Drafting the work or revising it critically for important intellectual content; NG, KH-L: Final approval of the version to be published.

### Conflict of interest statement

The authors declare that the research was conducted in the absence of any commercial or financial relationships that could be construed as a potential conflict of interest.
